# Efficacy and Safety of Denosumab in Osteoporosis or Low Bone Mineral Density Postmenopausal Women

**DOI:** 10.3389/fphar.2021.588095

**Published:** 2021-04-14

**Authors:** Yi Chen, Jun Zhu, Yiqin Zhou, Jinhui Peng, Bo Wang

**Affiliations:** Department of Orthopedics, Shanghai Changzheng Hospital, Naval Medical University, Shanghai, China

**Keywords:** denosumab, osteoporosis, bone mineral density, postmenopausal women, efficacy, safety, meta-analysis

## Abstract

Denosumab, a human monoclonal antibody, acts against the receptor activator of nuclear factor-κB ligand and is a promising antiresorptive agent in patients with osteoporosis. This study aimed to update the efficacy and safety of denosumab vs. placebo in osteoporosis or low bone mineral density (BMD) postmenopausal women. PubMed, Embase, Cochrane library, and ClinicalTrials.gov were searched for randomized controlled trials (RCTs) reporting the efficacy and safety data of denosumab vs. placebo in osteoporosis or low BMD postmenopausal women. A random-effects model was used to calculate pooled weight mean differences (WMDs) or relative risks (RRs) with corresponding 95% confidence intervals (CIs) for treatment effectiveness of denosumab vs. placebo. Eleven RCTs including 12,013 postmenopausal women with osteoporosis or low BMD were preferred for the final meta-analysis. The summary results indicated that the percentage change of BMD in the denosumab group was greater than that of BMD in placebo at 1/3 radius (WMD: 3.43; 95%CI: 3.24–3.62; *p* < 0.001), femoral neck (WMD: 3.05; 95%CI: 1.78–4.33; *p* < 0.001), lumbar spine (WMD: 6.25; 95%CI: 4.59–7.92; *p* < 0.001), total hip (WMD: 4.36; 95%CI: 4.07–4.66; *p* < 0.001), trochanter (WMD: 6.00; 95%CI: 5.95–6.05; *p* < 0.001), and total body (WMD: 3.20; 95%CI: 2.03–4.38; *p* < 0.001). Moreover, denosumab therapy significantly reduced the risk of clinical fractures (RR: 0.57; 95%CI: 0.51–0.63; *p* < 0.001), nonvertebral fracture (RR: 0.83; 95%CI: 0.70–0.97; *p* = 0.018), vertebral fracture (RR: 0.32; 95%CI: 0.25–0.40; *p* < 0.001), and hip fracture (RR: 0.61; 95%CI: 0.37–0.98; *p* = 0.042). Finally, denosumab did not cause excess risks of adverse events. These findings suggested that postmenopausal women receiving denosumab had increased BMDs and reduced fractures at various sites without inducing any adverse events.

## Introduction

Osteoporosis is defined as a bone disease that is characterized by decreased bone mineral density (BMD) and deteriorated micro-architecture of the skeleton, causing fragile bones and fracture risk(Brown et al., 2006). Osteoporosis is common, with a prevalence of 20–40% in postmenopausal women and 6–8% in men ≥50ºyears of age (Dubois et al., 2002; Sharma et al., 2008; Hernlund et al., 2013; Lee et al., 2013; Cosman et al., 2014; Institute for Clinical Systems Improvement (ICSI), 2017). It is more common in postmenopausal women, persons ≥65ºyears of age, Caucasians, Asians, and persons with a small body frame (Cosman et al., 2014; Institute for Clinical Systems Improvement (ICSI), 2017). Osteoporosis can occur as part of the aging process or secondarily due to nutritional deficiency, metabolic disorders, or medication side effects (National Library of Medicine, 2010; Cosman et al., 2014). Certain endocrine, gastrointestinal, hematologic, autoimmune, and central nervous system (CNS) disorders increase the risk of osteoporosis. Medications such as long-term anticoagulation (Caraballo et al., 1999; Gage et al., 2006), hormonal therapies (Tit et al., 2017; Tit et al., 2018), glucocorticosteroids (Etminan et al., 2008; Loke et al., 2011), some immunosuppressants (Anastasilakis et al., 2019), lithium (Zamani et al., 2009), thiazolidinediones (glitazones) (Majumdar et al., 2016), selective serotonin reuptake inhibitors (Diem et al., 2007; Richards et al., 2007), and long-term proton pump inhibitor use (Ngamruengphong et al., 2011; Khalili et al., 2012) may also cause osteoporosis(Cosman et al., 2014; Institute for Clinical Systems Improvement (ICSI), 2017). Osteoporosis accounts for greater than 90% of hip and vertebral fractures in women aged 65–84ºyears (NIH Consensus Development Panel on Osteoporosis Prevention, Diagnosis, and Therapy, 2001), utilizing major health services worldwide (Prevention and management of osteoporosis, 2003; Burge et al., 2007). With increase in the average life span, postmenopausal osteoporosis is becoming a serious public health issue in China and in many other countries. Moreover, osteoporotic fracture causes permanent disability, admission to institutional care, and even death (Sambrook and Cooper, 2006).

Currently, lifestyle recommendations (vitamin D and calcium supplementation, exercise, and smoking alcohol cessation) and antiresorptive agents as standard therapies for osteoporosis, with bisphosphonates as first-line treatment, were proved to have beneficial effects on BMD and risk of fragile fractures in postmenopausal women (Adachi et al., 2009; Clarke, 2009; Holick et al., 2011; Bauer, 2013; Cosman et al., 2014; Institute for Clinical Systems Improvement (ICSI), 2017; Qaseem et al., 2017). However, fracture occurs if the treatment strategies did not yield adequate response. Therefore, additional effective treatment agents should be identified to improve the prognosis of osteoporosis.

With advances in bone physiology, RANKL has already been identified as an important bone remodeling mediator. Moreover, RANK, as a receptor of RANKL, is observed during several stages of differentiation on osteoclast surface (Boyle et al., 2003; Hofbauer and Schoppet, 2004). The RANKL–RANK interaction is controlled by the soluble cytokine receptor, osteoprotegerin, which sequesters RANKL and neutralizes its effects (Simonet et al., 1997; Lacey et al., 1998).

Denosumab, a fully human monoclonal IgG2 antibody, binds to RANK ligand and affects the formation, function, and survival of osteoclasts (Fuller et al., 1998; Yasuda et al., 1998; Lacey et al., 2000). Several randomized controlled trials (RCTs) have been conducted for evaluating the treatment efficacy and safety of denosumab in osteoporosis or low-BMD postmenopausal women. But the treatment effectiveness of denosumab should be summarized to report the magnitude of effect estimates.

Therefore, this study aimed to investigate the treatment efficacy and safety of denosumab vs. placebo in osteoporotic or low-BMD postmenopausal women based on RCTs.

## Methods

### Data Sources, Search Strategy, and Selection Criteria

The Preferred Reporting Items for Systematic Reviews and Meta-Analysis Statement guidelines were used to conduct this study (Moher et al., 2009). PubMed, Embase, Cochrane library, and ClinicalTrials.gov were explored for published articles from inception till May 2019. The following search terms were used to retrieve the articles: osteoporosis, postmenopause, postmenopausal, women, denosumab, and randomized controlled trials. To obtain more appropriate and highly accurate studies, the reference lists of the obtained articles were also reviewed.

Two researchers selected the articles after initial screening. After that, careful screening of titles and abstracts of these articles was done. If the study was considered relevant, then full text of the study was obtained. Eligible studies should meet the following inclusion criteria (Brown et al., 2006): study design: studies designed as RCTs (Hernlund et al., 2013); patients: osteoporotic or low-BMD postmenopausal women (Lee et al., 2013); intervention: denosumab (Dubois et al., 2002); control: placebo (Sharma et al., 2008); outcomes: BMD or fracture at various sites, and any other potential adverse events. If several publications were available with increasing number of patients or longer follow-up for the same group, only data of 1- to 3-year follow-up duration were used for statistical analysis.

### Data Collection and Quality Assessment

The full-text and relevant data extraction from each study into the coding table in Microsoft Excel software was conducted by two reviewers. The following information including the first authors’ surname, publication year, country, sample size, mean age, body mass index (BMI), disease status, intervention and control, follow-up duration, and reported outcomes was extracted from each study. The JADAD scale was used to assess the quality of enrolled studies, which is based on randomization (1 or 0), concealment of the treatment allocation (1 or 0), blinding (1 or 0), completeness of follow-up (1 or 0), and the use of intention-to-treat analysis (1 or 0), and the scale system ranged from 0 to 5 (Jadad et al., 1996). The data collection and quality assessment were performed by two independent authors, and any conflicts between them were settled by an additional author by reviewing the original article.

### Statistical Analysis

Effect estimates for continuous data were presented as weighted mean differences with its 95% confidence intervals (CIs), while those of categorical data were expressed as relative risks (RRs) with corresponding 95% CIs. If the data in individual studies were expressed as median and range, then the data were converted to estimated means ± standard deviation before analysis. The summary effect estimates for efficacy and safety profiles were evaluated using the random-effects model (DerSimonian and Laird, 1986; Ades et al., 2005). Heterogeneity was evaluated across the included studies using I-square statistics and *p* value for Q statistics, and I-square greater than 50% or *p* < 0.10 was considered as significant heterogeneity (Higgins et al., 2003; Deeks et al., 2008). Sensitivity analysis was calculated to assess the impact of single individual trial from the overall analyses for clinical fractures (Pedroza-Tobías, 1999). Subgroup analyses for BMD and fractures were conducted based on the sites, and the treatment effects of denosumab among various sites were calculated using an interaction test (Altman and Bland, 2003). Publication bias for clinical fractures was evaluated using funnel plot (a pattern distribution roughly with the shape of a funnel indicates no publication bias), Egger (Egger et al., 1997), and Begg tests (Begg and Mazumdar, 1994). The inspective level for pooled results was 2-sided, and *p* < 0.05 was regarded as statistically significant. The analyses in this study were carried out through STATA software (version 12.0; Stata Corporation, College Station, TX, United States).

## Results

### Literature Search

**FIGURE 1:**
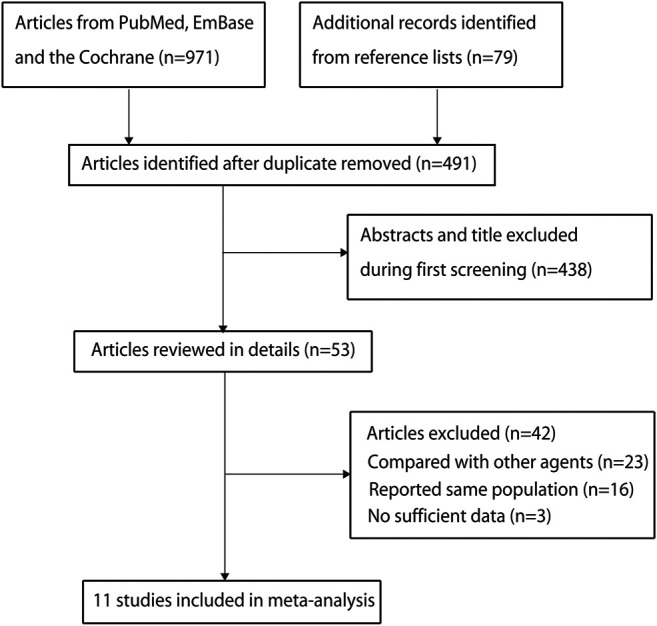
Flow diagram of study identification, and inclusion and exclusion criteria.

A preliminary initial search yielded 971 related records. After the titles and abstracts were reviewed, 918 studies were excluded due to duplications or irrelevant topics. For the remaining 53 articles, full texts were obtained and then reviewed. Of these, 42 were excluded for the following reasons: they used other control agents (*n* = 23), studies reported the same population (*n* = 16), and they were with no desirable outcomes (*n* = 3). Manual searching of the reference lists of the remaining studies yielded no additional study. Finally, 11 RCTs were selected for conducting this meta-analysis (Table 1; McClung et al., 2006; Bone et al., 2008; Ellis et al., 2008; Cummings et al., 2009; Seeman et al., 2010; Bone et al., 2011; Kumagai et al., 2011; Nakamura et al., 2012; Nakamura et al., 2014; Gnant et al., 2015; Koh et al., 2016). Figure 1 represents a flowchart of the selection process, and inclusion and exclusion criteria.

**TABLE 1:** Baseline characteristics of studies included in the meta-analysis.

Study	Publication year	Country	Sample size	Mean age (years)	BMI (kg/m^2^)	Disease status	Intervention	Follow-up duration, months	JADAD scale
McClung (AMG 162) Sambrook and Cooper (2006)	2006	United States	360	63.3	26.8	Lumbar spine BMD T score: 2.2; total hip BMD T score: 1.4; femoral neck BMD T score: 1.9; total body BMD T score: 1.4	Denosumab (6 mg, 14 mg, and 30 mg for 3 months; 14 mg, 60 mg, 100 mg, and 210 mg for 6 months); placebo	12.0	4
Bone Adachi et al. (2009)	2008	North America	332	59.4	26.4	Lumbar spine BMD T score: 1.61	Denosumab 60 mg every 6 months for 2 years; placebo	24.0	4
Ellis Bauer (2013)	2009	United States and Canada	252	59.4	27.8	Lumbar spine BMD T score: 1.06; total hip BMD T score: 0.95; femoral neck BMD T score: 1.27	Denosumab 60 mg every 6 months for 1 year; placebo	12.0 and 24.0	4
Cummings (FREEDOM) Holick et al. (2011)	2009	Multiple countries	7,808	72.3	26.0	Lumbar spine BMD T score: 2.83; total hip BMD T score: 1.90; femoral neck BMD T score: 2.16	Denosumab 60 mg every 6 months for 3 years; placebo	36.0	4
Seeman Clarke (2009)	2010	Argentina, Australia, Canada, France, and United States	165	60.5	NA	Lumbar spine BMD T score: 2.40; total hip BMD T score: 1.25	Denosumab 60 mg every 6 months for 1 year; placebo	12.0	5
Bone Qaseem et al. (2017)	2011	United States and Canada	256	59.1	26.7	Lumbar spine BMD T score: 1.61	Denosumab 60 mg every 6 months for 2 years; placebo	24.0	4
Kumagai Boyle et al. (2003)	2011	Japan	40	57.6	22.3	NA	Denosumab 0.03, 0.1, 0.3, 1.0, 3.0 mg/kg; placebo	9.0	3
Nakamura Hofbauer and Schoppet (2004)	2012	Japan	212	65.1	22.3	Lumbar spine BMD T score: 3.08; total hip BMD T score: 1.85	Denosumab 60 mg every 6 months for 1 year; placebo	12.0	4
Nakamura (DIRECT) Simonet et al. (1997)	2014	Japan	905	69.4	22.5	Lumbar spine BMD T score: 2.75; total hip BMD T score: 1.98; femoral neck BMD T score: 2.33	Denosumab 60 mg every 6 months for 3 years; placebo	36.0	4
Gnant (ABCSG-18) Lacey et al. (1998)	2015	Austria and Sweden	1,548	64.0	NA	Lumbar spine BMD T score: <-1.0	Denosumab 60 mg every 6 months for 2 years; placebo	24.0	5
Koh Fuller et al. (1998)	2016	Korea	135	66.5	23.6	Lumbar spine BMD T score: 2.95; total hip BMD T score: 1.95; femoral neck BMD T score: 2.45	Denosumab 60 mg every 6 months for 1 year; placebo	12.0	4

The JADAD scale was used to assess the quality of enrolled studies, which is based on randomization (1 or 0), concealment of the treatment allocation (1 or 0), blinding (1 or 0), completeness of follow-up (1 or 0), and the use of intention-to-treat analysis (1 or 0), and the scale system ranged from 0 to 5 (Jadad et al., 1996).

### Study Characteristics

Eleven RCTs recruited a total of 12,013 osteoporotic or low-BMD postmenopausal women. The follow-up duration for participants ranged from 9 to 36ºmonths, and 40–7,808 patients were included in each trial. The mean age of enrolled patients ranged from 57.6 to 72.3ºyears, and BMI ranged from 22.3 to 27.8ºkg/m^2^. Six studies were conducted in Western countries, four studies in Eastern countries, and the remaining one study in multiple countries. Two trials had a score of 5, eight trials had a score of 4, and the remaining one trial had a score of 3.

### Bone Mineral Density

**FIGURE 2:**
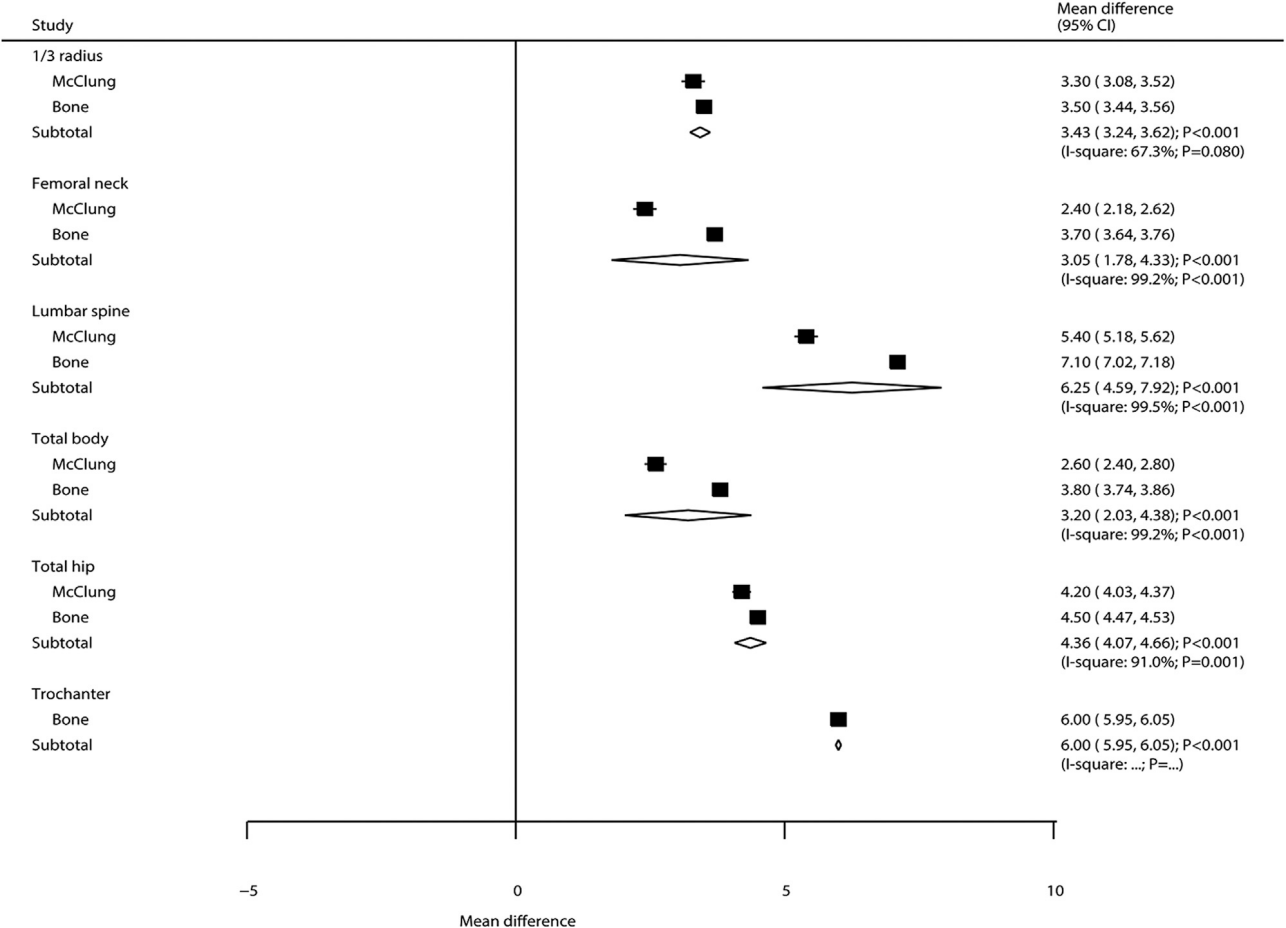
Summary results of BMD at various sites.

The summary results regarding the effectiveness of denosumab vs. placebo on BMD at various sites are presented in Figure 2. Overall, the results showed that the percentage of change in BMD was significantly increased with denosumab when compared with placebo at 1/3 radius (WMD: 3.43; 95%CI: 3.24 to 3.62; *p* < 0.001), femoral neck (WMD: 3.05; 95%CI: 1.78 to 4.33; *p* < 0.001), lumbar spine (WMD: 6.25; 95%CI: 4.59 to 7.92; *p* < 0.001), total hip (WMD: 4.36; 95%CI: 4.07 to 4.66; *p* < 0.001), trochanter (WMD: 6.00; 95%CI: 5.95 to 6.05; *p* < 0.001), and total body (WMD: 3.20; 95%CI: 2.03 to 4.38; *p* < 0.001). The included studies showed a significant heterogeneity for BMD at 1/3 radius, femoral neck, lumbar spine, total hip, and total body.

### Fracture

**FIGURE 3:**
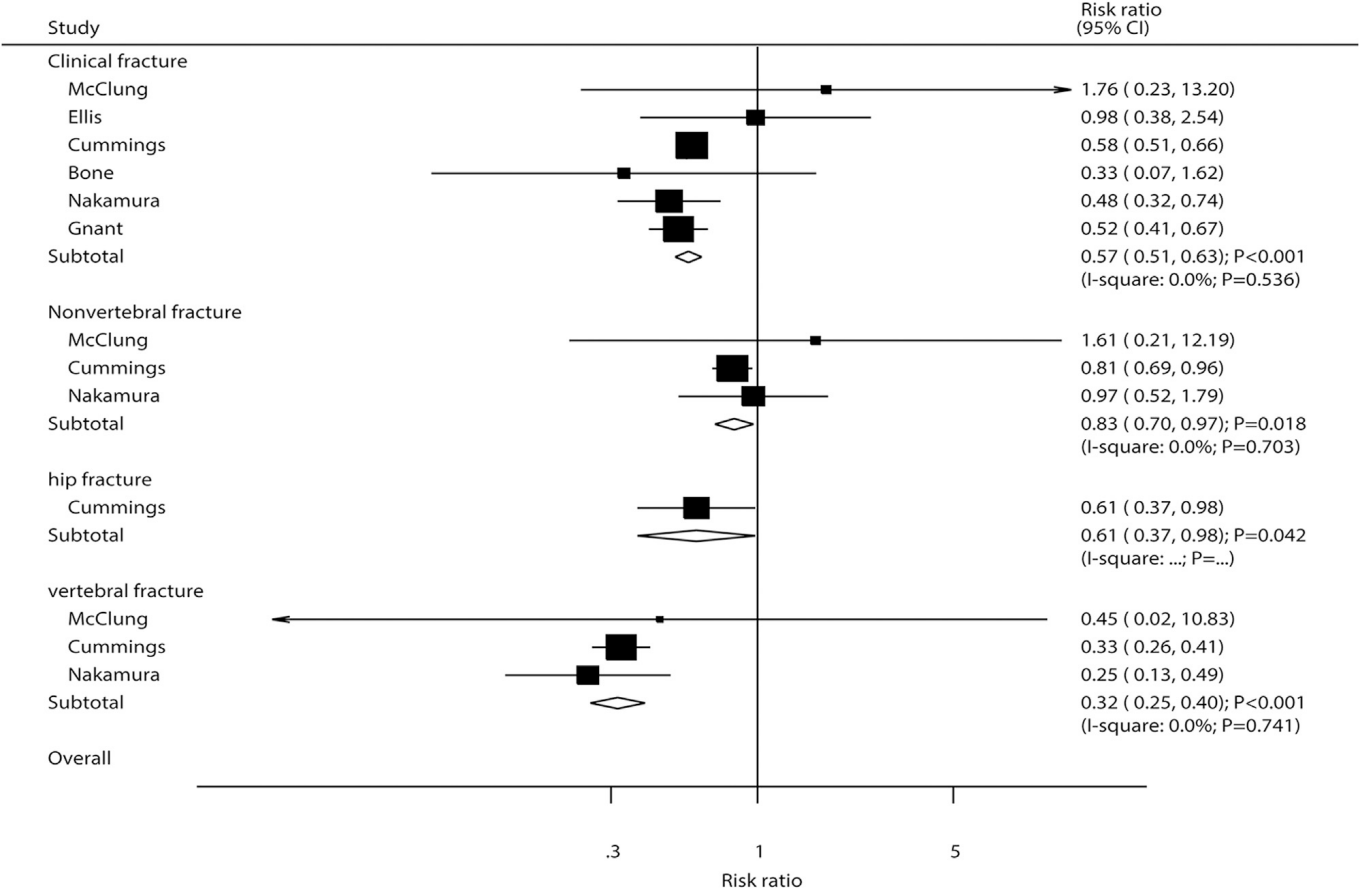
Summary results of fracture risk at various sites.

**FIGURE 4:**
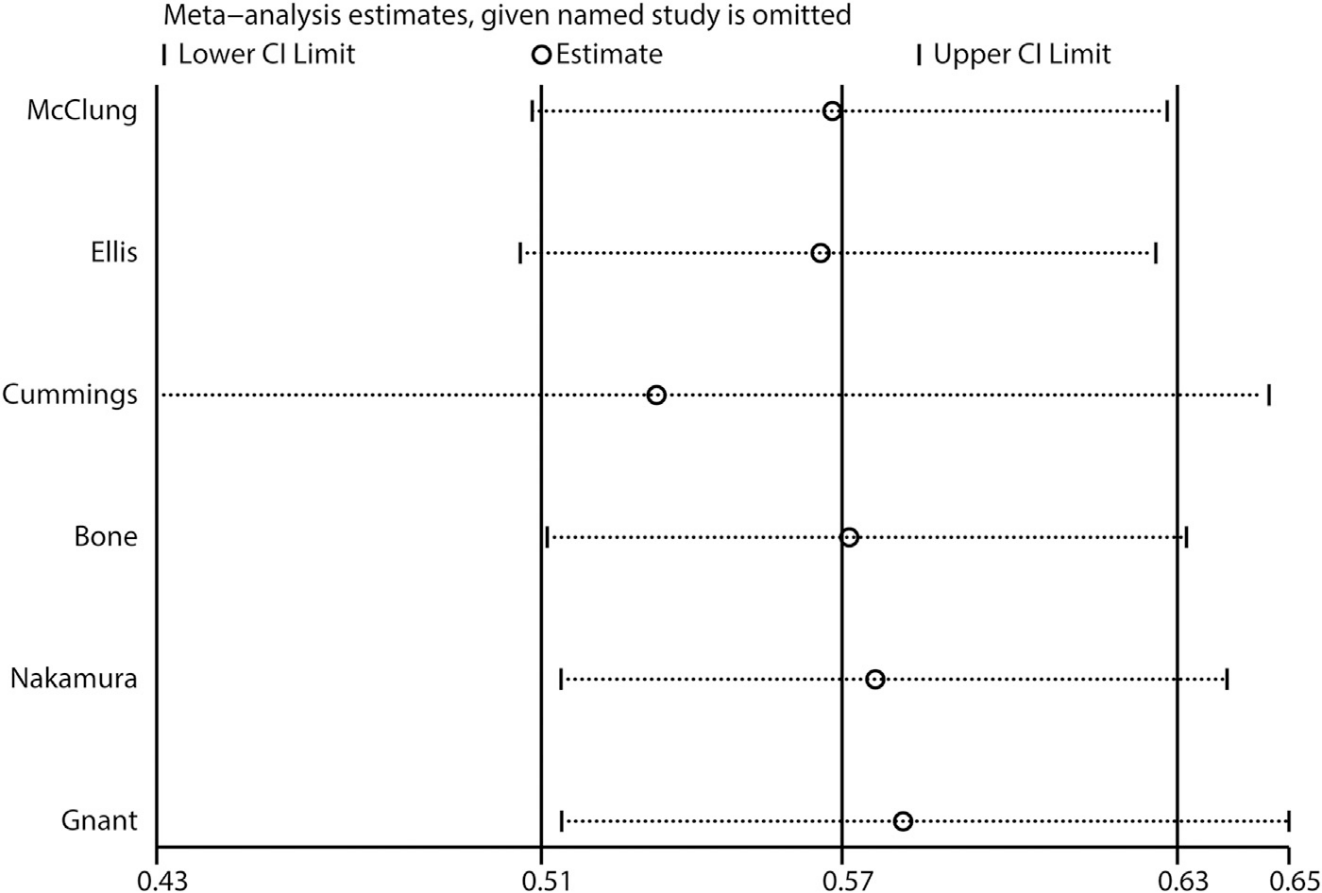
Sensitivity analysis of clinical fractures.

**FIGURE 5:**
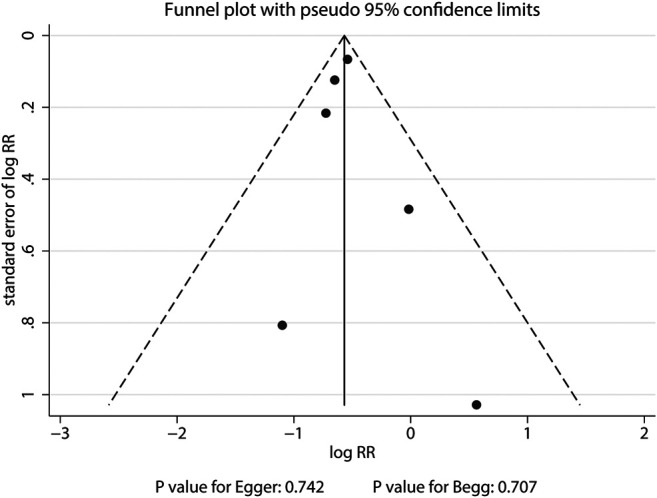
Funnel plot of clinical fractures. A pattern distribution roughly with the shape of a funnel indicates no publication bias.

The breakdown of the number of trials available for clinical fractures, nonvertebral fractures, vertebral fractures, and hip fractures was six trials, three trials, three trials, and one trial, respectively. The summary RRs indicated that the risk of clinical fractures (RR: 0.57; 95%CI: 0.51 to 0.63; *p* < 0.001), nonvertebral fractures (RR: 0.83; 95%CI: 0.70 to 0.97; *p* = 0.018), vertebral fractures (RR: 0.32; 95%CI: 0.25 to 0.40; *p* < 0.001), and hip fractures (RR: 0.61; 95%CI: 0.37 to 0.98; *p* = 0.042) was significantly reduced in patients who received denosumab (Figure 3). The included trials showed no heterogeneity for clinical fractures, nonvertebral fractures, and vertebral fractures. The results of sensitivity analysis indicated that the pooled conclusion for clinical fracture was stable and was unaltered by excluding any particular trial (Figure 4). Finally, no significant publication bias was detected through clinical fracture data (*p* value for Egger: 0.742; *p* value for Begg: 0.707; Figure 5).

### Safety Profiles

The summary results regarding the risk of adverse events are summarized in Supplementary Table S1. First, no significant differences were observed between denosumab and placebo for the risk of any adverse events (RR: 1.00; 95%CI: 0.99–1.01; *p* = 0.995; without evidence of heterogeneity), treatment-related adverse events (RR: 0.96; 95%CI: 0.75–1.23; *p* = 0.740; without evidence of heterogeneity), withdrawal due to adverse events (RR: 1.12; 95%CI: 0.84–1.48; *p* = 0.435; without evidence of heterogeneity), and death (RR: 0.80; 95%CI: 0.59–1.07; *p* = 0.137; without evidence of heterogeneity). Second, for adverse events that occur in at least 10% of subjects, complications such as constipation (RR: 1.53; 95%CI: 1.01–2.32; *p* = 0.043), flatulence (RR: 1.58; 95%CI: 1.12–2.22; *p* = 0.008), pharyngolaryngeal pain (RR: 3.02; 95%CI: 1.12–8.11; *p* = 0.029), and rash (RR: 3.00; 95%CI: 1.17–7.68; *p* = 0.022) were significantly increased in patients who received denosumab, whereas denosumab therapy was associated with low risk of falling (RR: 0.80; 95%CI: 0.66–0.97; *p* = 0.022) and periarthritis (RR: 0.17; 95%CI: 0.04–0.66; *p* = 0.010). No other significant difference was detected for any specific adverse events. Finally, denosumab and placebo showed no significant differences regarding the occurrence of serious adverse events or specific serious adverse events.

## Discussion

The prevalence of osteoporosis is on the rise and has become a serious public health issue of global concern, especially in postmenopausal women with advanced age, inducing greater fracture risk at various sites. The current study was conducted based on 11 RCTs with 12,013 osteoporosis or low-BMD postmenopausal women to evaluate the efficacy and safety of denosumab vs. placebo across a wide range of characteristics. The results of this study suggested that denosumab showed association with high percentage change in BMD at 1/3 radius, femoral neck, lumbar spine, total hip, trochanter, and total body. Moreover, the risk of various types of fractures such as clinical fractures, nonvertebral fractures, vertebral fractures, and hip fractures was significantly reduced in patients who received denosumab. Furthermore, denosumab did not yield additional risks on any adverse events, treatment-related adverse events, withdrawal due to adverse events, and deaths. Although denosumab and placebo treatments showed significant differences, these results might vary as fewer number of trials were included. As for homogeneity, the results from studies with greater weight were similar. Hence, even though the results from studies with lower weight were more variable, the overall results were still robust.

There are a large number of systematic reviews and meta-analyses conducted focusing on denosumab for treating postmenopausal women with osteoporosis or low BMD. Anastasilakis et al. (2009) conducted a meta-analysis of three RCTs and showed a significant decrease in the bone markers and increase of lumbar and hip BMD after treatment with denosumab, whereas no significant benefits were observed on the risk of fracture, and increased the infection risk. von Keyserlingk et al. (2011) conducted a meta-analysis based on four RCTs, and reported significant reduction in the risk of fracture without increasing adverse events in postmenopausal women who received denosumab. Zhou et al. (2014) carried out a meta-analysis of 11 RCTs and demonstrated a significant reduction in the risk of nonvertebral fractures and additionally yielded serious adverse events related to infection in osteoporotic or low-BMD postmenopausal women after treatment with denosumab. The meta-analysis conducted by Gu et al. (2015) was based on four RCTs, and the results revealed that the BMD was increased and the bone turnover markers were decreased in postmenopausal women after treatment with denosumab, whereas no significant risk for adverse events was observed. However, several RCTs have already been conducted regarding the topic, but should reevaluate the magnitude regarding the treatment effectiveness of denosumab vs. placebo in osteoporotic or low-BMD postmenopausal women. Therefore, the current quantitative meta-analysis was conducted to update the efficacy and safety of denosumab vs. placebo in postmenopausal women with osteoporosis or low BMD.

The summary results indicated that the percentage change of BMD in the denosumab group was greater than that of BMD in placebo at 1/3 radius, femoral neck, lumbar spine, total hip, trochanter, and total body, and these results were consistent with previous meta-analyses (Anastasilakis et al., 2009; Gu et al., 2015). Moreover, these results from individual trials reported similar conclusions, and the results of this study provided the magnitude of pooled results. Moreover, the current study suggested that the risk of clinical fractures, nonvertebral fractures, vertebral fractures, and hip fractures was significantly reduced in patients who received denosumab. The potential reasons for these conclusions could be due to denosumab action in inhibiting RANKL, preventing bone resorption, increasing BMD, and reducing fracture risk in osteoporosis or low-BMD postmenopausal women (Bekker et al., 2004; Lewiecki et al., 2007).

The summary results showed that denosumab therapy caused greater risk of constipation, flatulence, pharyngolaryngeal pain, and rash, whereas the risk of falling and periarthritis were significantly reduced. The potential reasons for this could be that denosumab could affect the immune system of the patients taking it and is associated with these adverse events (Canalis, 2010; Moen and Keam, 2011). Moreover, the risk of falling and periarthritis were reduced due to increased levels of BMD in osteoporotic or low-BMD postmenopausal women (Hita-Contreras et al., 2014; Burnett et al., 2017).

Although the current study provides comprehensive effectiveness results regarding denosumab in osteoporotic or low-BMD postmenopausal women, several limitations still existed and should be mentioned. First, the summary results for BMD at various sites were available only in few trials, inducing instability in the magnitude of BMD in the denosumab group. Second, the risk of most of the adverse events was available in smaller trials, and the power might not be enough to detect the difference between denosumab and placebo groups. Finally, publication bias remained inevitable since the analysis was conducted on published RCTs and that the bias against the publication of negative results is well known (Murad et al., 2018).

In conclusion, the findings of this meta-analysis indicated that osteoporotic or low-BMD women who received denosumab had increased BMD and reduced fracture risk at various sites. Moreover, the frequency of adverse events between denosumab and placebo groups was similar. Future large-scale RCTs that compare the treatment effectiveness of denosumab with other traditional drugs in patients at various stages should be conducted.
