# Supplementary material for: Efficacy and Safety of Denosumab in Osteoporosis or Low Bone Mineral Density Postmenopausal Women
Source: Front Pharmacol. 2021 Apr 14;12:588095. doi: 10.3389/fphar.2021.588095 (PMC8080120; doi:10.3389/fphar.2021.588095)
Supplement: Supplementary file 1 [file table1.docx]

Supplementary Table 1. The summary adverse events between denosumab and placebo

| **Outcomes** | **Number of trials** | **RR and 95% CI** | **P value** | **Heterogeneity (%)** | **P value for Heterogeneity** |
| --- | --- | --- | --- | --- | --- |
| Any adverse event | 10 | 1.00 (0.99-1.01) | 0.995 | 0.0 | 0.804 |
| Treatment-related adverse events | 5 | 0.96 (0.75-1.23) | 0.740 | 0.0 | 0.805 |
| Withdrawal due to adverse event | 5 | 1.12 (0.84-1.48) | 0.435 | 0.0 | 0.526 |
| Death | 4 | 0.80 (0.59-1.07) | 0.137 | 0.0 | 0.847 |
| **Adverse events occurring in at least 10% of subjects** | | | | | |
| Abdominal pain | 1 | 0.67 (0.11-3.89) | 0.652 | - | - |
| Arthralgia | 7 | 0.97 (0.87-1.07) | 0.525 | 0.0 | 0.732 |
| Back pain | 7 | 1.02 (0.86-1.21) | 0.822 | 0.0 | 0.809 |
| Bone pain | 1 | 1.23 (0.97-1.57) | 0.091 | - | - |
| Bronchitis | 1 | 0.82 (0.36-1.87) | 0.634 | - | - |
| **Constipation** | **4** | **1.53 (1.01-2.32)** | **0.043** | **0.0** | **0.617** |
| Contusion | 2 | 0.50 (0.11-2.23) | 0.365 | 65.7 | 0.088 |
| Cough | 1 | 2.42 (0.89-6.58) | 0.084 | - | - |
| Diarrhea | 2 | 0.60 (0.20-1.81) | 0.366 | 38.9 | 0.201 |
| Dyspepsia | 3 | 0.84 (0.41-1.73) | 0.639 | 0.0 | 0.574 |
| Eczema | 3 | 1.22 (0.69-2.18) | 0.496 | 82.9 | 0.003 |
| **Falling** | **1** | **0.80 (0.66-0.97)** | **0.022** | **-** | **-** |
| Fatigue | 2 | 1.06 (0.83-1.36) | 0.619 | 0.0 | 0.647 |
| **Flatulence** | **1** | **1.58 (1.12-2.22)** | **0.008** | **-** | **-** |
| Gastritis | 1 | 2.87 (0.31-26.90) | 0.356 | - | - |
| Gastroesophageal reflux | 2 | 1.25 (0.65-2.40) | 0.511 | 10.3 | 0.291 |
| Headache | 5 | 1.03 (0.70-1.50) | 0.898 | 0.0 | 0.596 |
| Hot flush | 1 | 1.13 (0.96-1.33) | 0.141 | - | - |
| Hypertension | 2 | 1.48 (0.48-4.54) | 0.494 | 26.1 | 0.245 |
| Influenza | 3 | 0.95 (0.60-1.51) | 0.839 | 1.6 | 0.362 |
| Insomnia | 1 | 0.80 (0.38-1.65) | 0.543 | - | - |
| Ligament sprain | 1 | 2.87 (0.31-26.90) | 0.356 | - | - |
| Myalgia | 1 | 3.83 (0.44-33.35) | 0.224 | - | - |
| Nasopharyngitis | 6 | 1.03 (0.82-1.30) | 0.809 | 0.0 | 0.636 |
| Nausea | 1 | 1.98 (0.49-8.04) | 0.341 | - | - |
| Pain in extremity | 5 | 1.20 (0.96-1.49) | 0.104 | 0.0 | 0.971 |
| **Periarthritis** | **1** | **0.17 (0.04-0.66)** | **0.010** | **-** | **-** |
| **Pharyngolaryngeal pain** | **1** | **3.02 (1.12-8.11)** | **0.029** | **-** | **-** |
| Potentially related to hypersensitivity | 1 | 0.87 (0.68-1.12) | 0.270 | - | - |
| **Rash** | **2** | **3.00 (1.17-7.68)** | **0.022** | **0.0** | **0.705** |
| Shoulder pain | 1 | 1.71 (0.81-3.62) | 0.161 | - | - |
| Sinusitis | 2 | 0.67 (0.36-1.27) | 0.222 | 0.0 | 0.528 |
| Upper abdominal pain | 1 | 0.63 (0.21-1.83) | 0.392 | - | - |
| Upper respiratory tract infection | 3 | 0.90 (0.55-1.49) | 0.686 | 32.4 | 0.228 |
| Urinary tract infection | 2 | 1.87 (0.29-12.19) | 0.513 | 52.7 | 0.146 |
| **Serious adverse event** | | | | | |
| All | 9 | 1.04 (0.96-1.11) | 0.336 | 0.0 | 0.586 |
| Abnormal clinical laboratory investigation | 1 | 0.45 (0.02-10.83) | 0.621 | - | - |
| Acute cholecystitis | 1 | 3.00 (0.12-72.60) | 0.499 | - | - |
| Amnesia | 1 | 0.33 (0.01-8.07) | 0.499 | - | - |
| Cancer | 1 | 1.15 (0.91-1.45) | 0.247 | - | - |
| Cardiac disorder | 5 | 1.02 (0.84-1.23) | 0.879 | 0.0 | 0.407 |
| Cholelithiasis | 1 | 0.20 (0.01-4.10) | 0.296 | - | - |
| Confusional state | 1 | 0.33 (0.01-8.07) | 0.499 | - | - |
| Ear and labyrinth disorders | 1 | 1.04 (0.04-25.10) | 0.982 | - | - |
| Endocrine disorders | 1 | 1.75 (0.89-3.44) | 0.105 | - | - |
| Eye disorders | 1 | 0.77 (0.46-1.30 | 0.330 | - | - |
| Gastrointestinal disorders | 3 | 1.16 (0.23-5.87) | 0.856 | 10.0 | 0.329 |
| General disorders | 1 | 0.75 (0.04-15.30) | 0.849 | - | - |
| Hepatobiliary disorders | 1 | 0.34 (0.01-8.17) | 0.502 | - | - |
| Hyperglycemia | 1 | 3.00 (0.12-72.60) | 0.499 | - | - |
| Infection | 6 | 1.14 (0.65-2.00) | 0.644 | 18.2 | 0.295 |
| Injury, poisoning, or procedural complication | 5 | 0.80 (0.57-1.12) | 0.194 | 0.0 | 0.720 |
| Loss of consciousnes | 1 | 3.00 (0.12-72.60) | 0.499 | - | - |
| Musculoskeletal or connective-tissue disorder | 5 | 1.08 (0.86-1.37) | 0.499 | 0.0 | 0.422 |
| Neoplasm | 5 | 0.94 (0.46-1.91) | 0.857 | 0.0 | 0.660 |
| Nervous system disorders | 6 | 1.07 (0.84-1.38) | 0.579 | 0.0 | 0.903 |
| Pneumonia | 1 | 0.33 (0.01-8.07) | 0.499 | - | - |
| Psychiatric disorders | 1 | 0.34 (0.01-8.17) | 0.502 | - | - |
| Reproductive system or breast disorder | 1 | 1.01 (0.06-15.95) | 0.997 | - | - |
| Vascular disorder | 2 | 0.91 (0.10-8.17) | 0.934 | 0.0 | 0.539 |
